# Interoceptive-autonomic pathways in mood state transitions in bipolar disorder: a hypothesis-generating brief review

**DOI:** 10.3389/fpsyt.2026.1996853

**Published:** 2026-10-30

**Authors:** Yahui Zhang, Jilei Wan, Jia Chen, Qingrui Li

**Affiliations:** 1 Department of Health Management, Aerospace Center Hospital, Beijing, China; 2 Department of Traditional Chinese Medicine, Aerospace Center Hospital, Beijing, China

**Keywords:** autonomic nervous system, bipolar disorder, circadian rhythm, emotion regulation, heart rate variability, interoception, predictive coding

## Abstract

Bipolar disorder is typically described in terms of clinical states such as depression, mania, or hypomania, but bodily signal processing, autonomic regulation, and emotion regulation across these states have not yet been integrated into a single testable model. This review distinguishes cross-sectional correlates of mood states from longitudinal predictors of subsequent mood changes. It proposes a state-dependent framework linking sleep–circadian disruption, autonomic flexibility, interoceptive processing, and emotion regulation; the temporal sequence and causal contributions of these components remain unestablished. Cross-sectional findings describe concurrent associations, recovery studies characterize changes accompanying symptom resolution, and prospective studies inform temporal ordering and prediction; none alone establishes a mechanism driving mood-state transitions. This article discusses direct evidence from bipolar disorder, transdiagnostic evidence, and theoretical inference in a hierarchical manner, without equating HRV with interoception or treating altered interoception as a single pathophysiological mechanism of bipolar disorder. Finally, it proposes a research protocol comprising state-stratified heartbeat discrimination/signal detection tasks, HRV reactivity and recovery, sleep and activity rhythms, ecological momentary assessment (EMA), and longitudinal assessment of state transitions.

## 1 Introduction

Patients with bipolar depression often report fatigue, bodily heaviness, gastrointestinal discomfort, and difficulty initiating action, whereas patients with mania or hypomania exhibit reduced need for sleep, psychomotor activation, and increased action impulsivity. Although the clinical presentations are diametrically opposed, the two states jointly point to a central question: Are transitions in mood states accompanied by dynamic changes in the perception, appraisal, and regulation of bodily signals?

Previous depression research has proposed that interoceptive dysfunction may serve as a bridge between somatic symptoms and affective disorders; however, whether this framework can be directly transferred to a state-specific model of bipolar disorder remains open to debate (1). Current systematic reviews and meta-analyses of heart rate variability (HRV) in bipolar disorder suggest that patient groups generally show lower HRV than healthy controls; however, heterogeneity across studies and confounders such as medication and lifestyle substantially weaken the robustness of these conclusions (2). The International Society for Bipolar Disorders (ISBD) Chronobiology Task Force has highlighted associations between rhythm disturbances and illness course, recurrence, and mood-state regulation; however, their temporal ordering and causal contributions remain incompletely established (3). Moreover, meta-analyses have confirmed that emotion regulation difficulties are prevalent in bipolar disorder (4).

Against this background, the central question of the present study is not to broadly verify the association between bipolar disorder and interoception, but to examine whether mood state transitions, interoceptive assessment, autonomic flexibility, and sleep-circadian rhythm disturbances can be integrated into an empirically testable temporal model. Although these dimensions have been examined separately, their integration within a unified framework of mood-state transitions remains insufficiently investigated. Here, mood-state transitions refer to changes between euthymia and mood episodes or between episode polarities. Continuous symptom changes, episode relapse, and polarity switching are distinguished when interpreting individual studies. Throughout this review, cross-sectional associations are interpreted as concurrent correlates, without assuming state specificity or temporal precedence. Within-person longitudinal changes inform state dependence, and prospective associations inform prediction; neither alone establishes a causal mechanism driving mood-state transitions.

This narrative mini-review was informed by preliminary, purposive PubMed searches completed by September 4, 2026, to scope the topic and assess novelty. Searches addressed mood disorders, interoception, emotion regulation, and autonomic function/HRV. Literature on sleep/circadian rhythms, wearable monitoring, emotional processing, and predictive coding was supplemented during manuscript development, without complete prospective documentation of these supplementary searches. Publications were selected for their conceptual and methodological relevance, and evidence was synthesized narratively, distinguishing bipolar-specific findings, transdiagnostic evidence, and theoretical inference. No prespecified eligibility criteria, duplicate independent screening, or formal risk-of-bias assessment were applied; the cited literature represents evidence selected for theory development rather than a systematically identified evidence base.

## 2 Interoception, autonomic function, and emotion regulation: concepts and research evidence

### 2.1 Deconstructing the multidimensional structure of interoception: accuracy, sensibility, decision criteria, and attribution

Clarifying the multidimensional structure of interoception is a prerequisite for examining its role in bipolar state transitions. Research on interoception should at minimum distinguish among objective accuracy, subjective sensibility, response bias, and bodily attribution. Where the task permits, signal detection indices distinguish perceptual sensitivity (d′) from response criterion (c) (5). Questionnaires assess subjective interoceptive sensibility and should be identified by instrument and subscale rather than treated as measures of objective accuracy. A transdiagnostic task-based fMRI meta-analysis indicated that patients with various psychiatric disorders exhibit convergent aberrant activation in the left dorsal mid-insula during interoceptive processing; however, because the study pooled different diagnoses and tasks, the statistical power for disorder subgroup and activation direction analyses was insufficient, and therefore no inference can be drawn regarding differences among mood states in bipolar disorder (6). The distinction proposed by Garfinkel et al. among interoceptive accuracy (performance on objective behavioral tests of heartbeat detection), interoceptive sensibility (self-assessed subjective interoceptive ability via interviews/questionnaires), and interoceptive awareness (metacognitive awareness of interoceptive accuracy, such as confidence–accuracy correspondence) provides multiple measurement dimensions for interpreting findings in bipolar disorder (7). The predictive coding model conceptualizes interoception as a continuous comparison between predictions of bodily states and afferent signals; on this basis, the model links the sense of presence with the sense of agency, the core idea being that parallel predictive coding mechanisms determine whether incoming sensory signals are internally generated or externally caused, and the predicted interoceptive signal depends on the outcome of this determination (8, 9). Therefore, the same low self-report score may arise from reduced sensory precision, diminished bodily attention, altered decision criteria, or negative attribution, rather than from a unitary “interoceptive deficit.” Some studies also support viewing interoception as a common risk dimension for psychopathology rather than a diagnosis-specific marker (10). Thus, interoception is not a unitary variable; studies of bipolar disorder must differentiate among measurement dimensions and should not uniformly interpret low self-report scores as interoceptive deficits.

### 2.2 Interoception research in bipolar disorder: current evidence and limitations

Jialin Lv et al. conducted a cross-sectional study of 71 young people aged 14–25 years with bipolar disorder in the maintenance phase and 111 age-matched controls. The results showed that interoceptive sensibility was significantly lower in patients with bipolar disorder than in controls, and that certain dimensions of bodily attention (such as not distracting, not worrying, self-regulation, and trust) were significantly correlated with somatic and emotional symptoms. Patients with somatic symptoms had the highest depression and anxiety scores and the lowest scores on the above interoceptive dimensions. In cross-sectional regression analyses, the ‘not distracting’ dimension remained independently associated with somatic and emotional problems after adjustment for mood symptoms; this association does not establish prospective prediction or temporal precedence (11). These findings suggest that interoception may be associated with psychosomatic symptoms in young people with bipolar disorder; however, given the limited research in this area, the independent contribution of each interoceptive dimension to somatic symptoms and their patterns of association with mood symptoms remain unclear (11).

Gaspare Alfì et al. assessed interoceptive sensibility using the Multidimensional Assessment of Interoceptive Awareness (MAIA) in 60 inpatients with bipolar II depression and 36 controls. Patients showed significantly lower interoceptive sensibility than controls, and interoceptive sensibility was negatively correlated with emotion regulation difficulties. Cross-sectional mediation analysis identified a statistically significant indirect association involving interoceptive sensibility, but did not establish temporal ordering or causal mediation (12). These studies identify concurrent associations involving self-reported interoceptive sensibility, but do not establish mood-state specificity or distinguish state-dependent changes from stable individual differences. They also do not show that interoceptive alterations precede or drive mood-state transitions; reverse causation, medication effects, illness burden, and shared determinants remain plausible explanations. These findings therefore motivate hypotheses rather than establish a directional mechanism.

### 2.3 Heart rate variability as an indicator of autonomic function: evidence and limitations research on HRV in bipolar disorder has accumulated considerable evidence, but studies differ substantially in methodological heterogeneity, definition of mood states, and control of confounders

An opinion article focusing on bipolar II disorder proposed that autonomic regulation may serve as a candidate physiological buffering process linking somatic states and mood stability, although this hypothesis still requires empirical testing (13). A study by Abigail Ortiz et al. found that lower HRV levels were associated with a higher illness burden in bipolar disorder (14). Corponi et al. repeatedly assessed HRV from acute bipolar episodes to euthymia and reported evidence of increasing lnRMSSD during symptom resolution (15). These findings indicate within-person HRV changes accompanying recovery, but do not establish that these changes precede or drive recovery or subsequent mood-state transitions. Testing temporal precedence requires repeated within-person assessments before episode onset, prespecified and clinically adjudicated episode criteria, and adequate control of medication, respiration, physical activity, and substance use; establishing a causal mechanism requires additional evidence. Critical reviews have also noted that the relationship between HRV and mood states is significantly influenced by respiratory rate, measurement conditions, and analytical methods (16). HRV quantifies variation in normal-to-normal cardiac intervals. RMSSD is the root mean square of successive interval differences, and lnRMSSD is its natural logarithm. Under standardized conditions, RMSSD primarily indexes cardiac vagal modulation rather than overall autonomic function or interoception. Here, autonomic flexibility is operationalized through reactivity and recovery in a prespecified HRV index. Reactivity denotes change from baseline during a defined challenge, whereas recovery denotes the post-challenge trajectory toward baseline. The challenge, recording windows, and calculation methods should be specified. Recording duration, body posture, respiratory monitoring and any respiratory control procedures, and artifact correction methods should be reported (17).

### 2.4 Emotion regulation abnormalities in bipolar disorder: neural basis and clinical manifestations

Emotion recognition concerns identifying others’ emotions from expressive cues (18), whereas emotional clarity concerns understanding one’s own feelings (19). Alexithymia involves difficulties identifying and describing feelings and externally oriented thinking (20). Emotion regulation concerns processes shaping emotional experience and expression (21). These constructs are related but non-equivalent. Emotion labelling refers here to naming one’s own feelings. A review of the functional neuroanatomy of mania indicated that reward, salience, and prefrontal control networks jointly participate in hyperactivated states (22). Katharina Förster et al., based on a meta-analysis of emotional processing across bipolar states, found that neural responses to positive and negative emotions are not uniform across states; compared with healthy controls, patients with bipolar disorder show increased activation in brain regions involved in the salience processing of emotional stimuli (such as the bilateral insula) and decreased activation in emotion regulation-related regions (such as the inferior frontal gyrus) during emotional processing. More detailed descriptive analyses revealed that patients with bipolar disorder in manic episodes show decreased anterior insula activation when processing negative emotions (relative to positive emotions) (23). Systematic reviews and meta-analyses report difficulties in emotion regulation and alterations in its neural correlates in bipolar disorder (24–26). Comparative meta-analysis indicates that emotion dysregulation is shared across psychiatric disorders, with differences between bipolar disorder and other diagnoses varying by the dimension assessed (27). A separate meta-analysis reports associations between emotion dysregulation and depressive or (hypo)manic symptoms within bipolar disorder (28). Mesbah et al.’s meta-analysis identified altered task-related activation, predominantly within fronto-limbic regions, in bipolar disorder (29). Emily S Gillissie et al.’s meta-analysis of social cognition indicates that the recognition and interpretation of emotional cues may also be impaired (30). These findings concern social-emotional cue processing and do not directly establish impaired identification or clarity of one’s own feelings. The above evidence suggests the presence of emotion regulation vulnerability in bipolar disorder, but whether interoceptive abnormalities constitute an upstream mechanism remains to be verified. A more cautious interpretation is that interoception may participate in the transformation of bodily signals into emotional and behavioral outputs.

## 3 Sleep and circadian rhythm disturbances: potential contributing factors to mood state transitions

The preceding discussions of interoception and autonomic function have focused on the “encoding and transformation of bodily signals,” whereas sleep and circadian research provides prospective evidence relevant to temporal ordering, although temporal precedence should be distinguished from causal influence. Circadian rhythm disturbances are widespread in bipolar disorder, but their relationship with mood state transitions remains unclear. Ni Xu et al., through a systematic review and meta-analysis, found that evidence regarding the effects of lithium on circadian phenotypes in bipolar disorder is insufficient (31). Jan Scott et al., based on a systematic review and meta-analysis in populations at high risk for or with early-onset bipolar disorder, found that sleep and circadian rhythm disturbances are important risk signals for bipolar disorder (32). Building on this, Esaki et al. found that baseline circadian activity characteristics were prospectively associated with subsequent mood-episode relapse over 12 months. This supports prognostic relevance, but does not establish a causal pathway through autonomic or interoceptive changes (33). However, research on bipolar I disorder indicates that whether rhythm abnormalities drive transitions toward hyperactivated or hypoactivated states remains a hypothesis to be tested rather than an established conclusion (34).

Longitudinal wearable studies provide evidence relevant to temporal ordering and prediction. Using model-estimated circadian phase and transfer entropy analysis, Song et al. reported directional relationships from circadian phase disturbances to subsequent daily mood symptoms in major depressive disorder and bipolar I disorder, but not bipolar II disorder (35). These findings concern daily symptom dynamics rather than directly demonstrating mechanisms initiating clinically defined mood episodes. Lim et al. developed a model predicting next-day mood-episode status from wearable-derived sleep and circadian features in a mixed mood-disorder cohort (36). Together, these observational findings support temporal or predictive relationships, but do not establish that modifying these features prevents transitions or that autonomic–interoceptive changes mediate their associations. Frederike T Fellendorf et al. demonstrated the feasibility of daily data collection through smartphone-based sleep monitoring studies (37). Luigi F Saccaro et al., in a systematic review of digital phenotyping, proposed that new technologies provide valuable opportunities for digital phenotyping with objectivity and high precision, and that artificial intelligence-based models are expected to provide important support for clinical decision-making (38). However, limitations such as patient adherence, missing data, device differences, and algorithmic extrapolation must be incorporated into research reports when analyzing data from digital devices (38). Even during remission, differences in wrist actigraphy between lithium and other mood stabilizers cannot be directly interpreted as interoceptive differences (39).

## 4 An integrative hypothesis of mood state transitions

### 4.1 Overall pathway: from sleep and circadian rhythm disturbances to emotion regulation

The proposed model hypothesizes that sleep/circadian disturbances precede changes in bodily-state prediction and autonomic flexibility, followed by changes in interoceptive processing and emotion regulation (**Figure 1**). This sequence remains unverified; concurrent mood symptoms, reverse directionality, and shared determinants remain alternative explanations. Evidence differs across links. Baseline circadian activity features show prospective associations with mood-episode relapse in bipolar disorder (33), whereas sleep and model-estimated circadian features have shown predictive value for next-day episode status in a mixed mood-disorder cohort (36). Transfer entropy analyses suggest directional relationships from model-estimated circadian phase to daily mood symptoms in major depressive disorder and bipolar I disorder, with no comparably clear evidence in bipolar II disorder (35). The proposed links from sleep/circadian disturbances to bodily-state prediction and autonomic flexibility are theoretical extensions informed by interoceptive predictive coding frameworks (8, 9) and an autonomic buffering hypothesis (13). These links have not been directly established in bipolar disorder, and reciprocal influences remain possible. The reviewed HRV findings do not directly establish autonomic–interoceptive coupling or its temporal and causal direction in bipolar disorder. Cross-sectional evidence links self-reported interoceptive sensibility to emotion regulation difficulties in bipolar II depression (12), while studies in healthy participants (40) and patients with depression/anxiety disorders (41) report associations between self-reported interoceptive dimensions and emotion regulation strategy use. These findings support associations rather than establish a directional mechanism in bipolar disorder. The proposed intervening roles of emotion labelling and emotional clarity, their links to state-specific behavioral outputs, and the proposed feedback to sleep/circadian disruption remain unverified within this integrated model.

**Figure 1:**
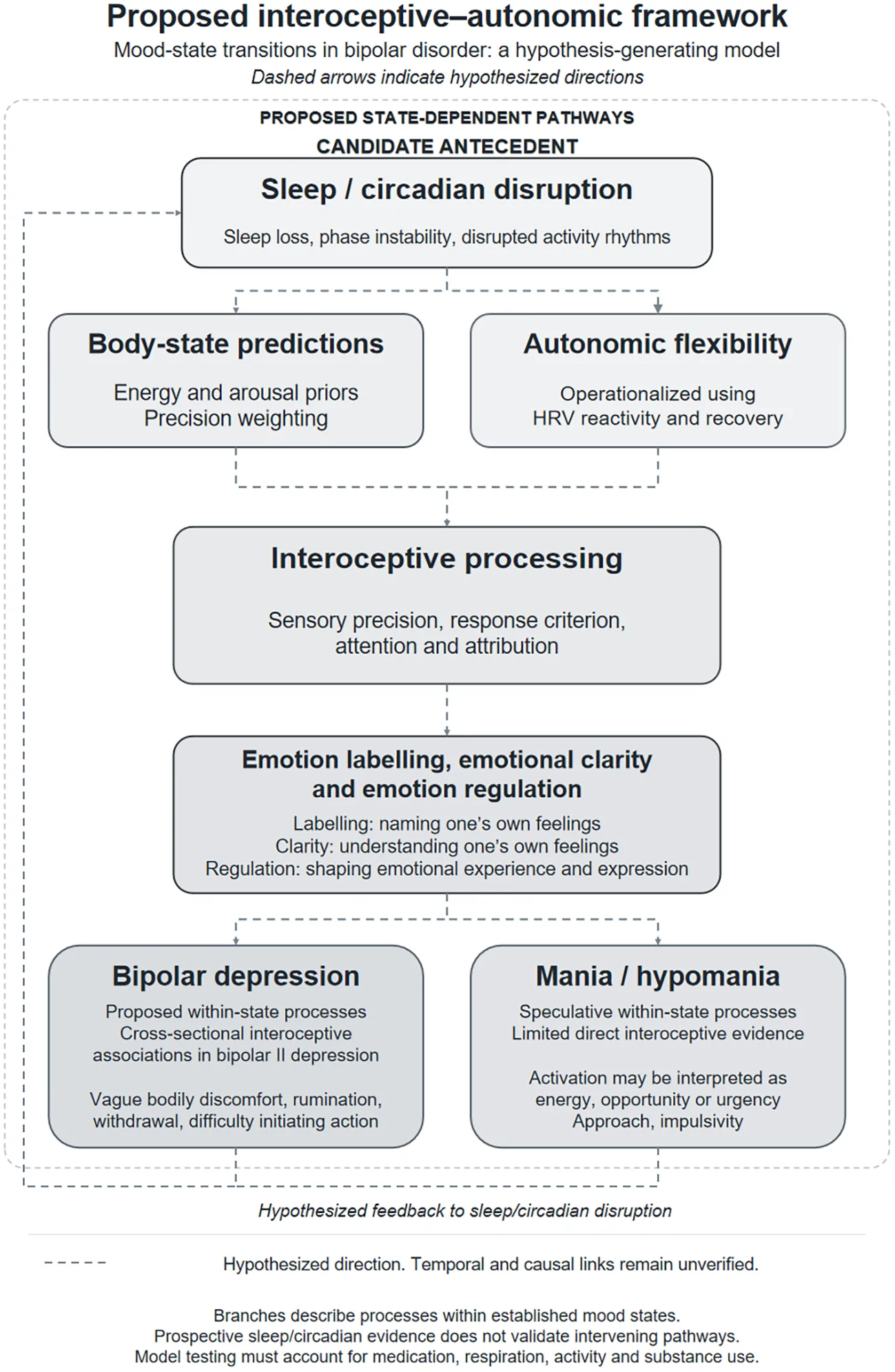
Proposed interoceptive–autonomic framework for mood-state transitions in bipolar disorder. The diagram presents a hypothesis-generating framework, with sleep and circadian disturbances positioned as candidate antecedents. Dashed arrows indicate hypothesized directions linking body-state predictions, autonomic flexibility, interoceptive processing, emotion labelling, emotional clarity, and emotion regulation. These directions do not represent established temporal or causal pathways, and reciprocal influences remain possible. Autonomic flexibility is operationalized using HRV reactivity and recovery. Emotion labelling, emotional clarity, and emotion regulation are related but non-equivalent constructs grouped for presentation, without implying an established sequence among them. The depressive branch and the branch for mania and hypomania describe proposed processes within established mood states rather than demonstrated mechanisms initiating transitions. Cross-sectional findings in bipolar II depression support associations between self-reported interoceptive sensibility and emotion regulation difficulties, but do not validate the depressive pathway. The interoceptive pathway in mania and hypomania remains particularly speculative. The outer dashed pathway represents hypothesized feedback from the mood-state branches to sleep and circadian disturbances. Prospective and predictive findings on sleep and circadian rhythms do not establish the intervening autonomic–interoceptive pathways. Medication, respiration, physical activity, substance use, and other relevant covariates require consideration when testing the model.

This model does not assume that all components are abnormal in every patient, nor does it treat interoceptive measures as direct causes of the disorder. Coll et al.’s systematic review and meta-analysis reported an association between heartbeat-evoked potential amplitude and behavioral measures of cardiac interoception. However, variation in measurement protocols and potential cardiac and somatosensory confounds limit interpretation of this association (42). An everyday experience sampling study by Giulia L Poerio et al. showed dynamic relationships between interoceptive attention and current emotional states, for example, relationships between interoceptive attention and emotions in daily life that are specific to interoceptive attention (43). Transdiagnostic research in clinical service populations suggests that cardiac interoception may show heterogeneity within mental health samples (44). Some studies indicate that difficulties in emotion naming may be linked to different dimensions of interoception rather than to a simple sensory deficit (45).

### 4.2 Hypothesized interoceptive processes in bipolar depression

During depressive episodes, low energy, disrupted sleep, and negative internal states may reduce the efficiency with which bodily signals are matched to emotion categories. Within this hypothesis, bodily heaviness, fatigue, or gastrointestinal discomfort may be experienced as poorly differentiated discomfort and may be associated with rumination, withdrawal, and difficulty initiating action. This hypothesized pathway may involve one or more of the following components: interoceptive attention, decision criteria, sensory precision, or negative attribution; current evidence can only support a testable hypothesis, not a single mechanism. This account concerns processes during an established depressive episode; whether they precede episode onset, follow symptom emergence, or contribute to episode persistence remains unresolved.

### 4.3 Hypothesized interoceptive processes in mania or hypomania

During manic or hypomanic episodes, reduced sleep need and hyperactivation may increase the salience of action-related bodily predictions, potentially influencing how psychomotor activation is interpreted as energy, opportunity, or urgency. If recovery processes and reflective monitoring are insufficient, behavioral output may become biased toward approach, impulsivity, and persistent goal pursuit. Evidence on the functional neuroanatomy of mania and emotional processing provides background context but does not directly test this hypothesized interoceptive pathway (22, 23), which remains speculative. This account concerns processes during established mania/hypomania and does not demonstrate an antecedent mechanism of switching into these states.

### 4.4 Potential role of emotion regulation between interoception and clinical manifestations

Yafei Tan et al. used experience sampling methods to investigate the role of interoceptive attention in the dynamic changes of emotion regulation (ER) strategies, and found associations between interoceptive attention and emotion regulation strategy use in healthy participants (40). Su Jin Lee et al. also found associations among interoceptive awareness, emotion regulation, and symptom severity in studies of depression, anxiety, and somatization (41). These non-bipolar findings support associations relevant to the model, but do not establish causal direction or mediation in bipolar disorder. Arnaldo et al. proposed an active inference account linking allostatic overload to depression (46). The “interoceptive model of energy allostasis” proposed by Sara Z Mehrhof provides a broader theoretical background for mental health linking metabolic signals, homeostasis, and psychological well-being (47).

## 5 Testable research questions and future study designs

RQ1: State differences. Do bipolar depression, mania/hypomania, euthymia, and healthy controls show replicable differences in interoceptive accuracy, sensibility, response bias, HRV reactivity, and recovery?

RQ2: Coupling mechanisms. Does autonomic flexibility moderate the relationship between difficulties identifying one’s own feelings and interoceptive judgment bias? Does this moderation change across states?

RQ3: Temporal order. Do within-person sleep/circadian changes precede autonomic–interoceptive changes, and do these changes predict subsequent mood-state transitions beyond prior symptom severity and time-varying confounders? Are reverse temporal pathways also supported?

Priority designs should include:

Repeated measures within the same participants across depressive, manic/hypomanic, and euthymic states.

Heartbeat discrimination/signal detection tasks that distinguish perceptual sensitivity from response criterion.

Synchronized sampling of ECG/HRV, respiration, electrodermal activity, and activity.

EMA, actigraphy, and sleep diaries, with documentation of medication class, dose and timing, respiration, recent physical activity, and substance use, including caffeine, nicotine and alcohol. Relevant covariates, including age, sex, body mass index, cardiovascular conditions, sleep and recording time, should be standardized where feasible or addressed in prespecified analyses. Multilevel longitudinal models with state transitions as outcomes, rather than merely comparing group means.

Experimental manipulation of candidate factors, where feasible, with temporally ordered assessments of downstream autonomic, interoceptive, and mood outcomes to test proposed causal links. The evidence and testable next steps across the interoceptive, autonomic, sleep/circadian, and emotion-regulation dimensions are summarized in **Table 1**.

**Table 1:** Evidence for interoception–autonomic research in bipolar disorder.

Dimension	Evidence and citations	Evidence and testable next steps
Interoceptive constructs and measurement	Cross-sectional self-report studies in young people with bipolar disorder (11) and inpatients with bipolar II depression (12) provide evidence of associations. Measurement distinctions, predictive coding models, and transdiagnostic reviews provide conceptual and methodological frameworks (7–10).	Cross-sectional self-report associations do not establish temporal precedence or causal mediation. Repeated within-person assessments are needed to help distinguish mood-state-related variation from stable individual differences.
Autonomic flexibility	HRV meta-analysis and illness-burden studies provide evidence of associations in bipolar disorder (2, 14), while repeated assessments characterize changes accompanying recovery (15). A perspective article proposes a candidate buffering hypothesis (13), and a critical review provides broader methodological context (16).	HRV differences and changes accompanying recovery do not establish a mechanism that precedes mood-episode onset. Prospective measurements before episode onset are needed to assess whether HRV changes precede mood episodes.
Sleep and circadian rhythms	ISBD task force reviews, lithium meta-analyses, reviews of high-risk populations, relapse cohorts, and state-transition studies constitute evidence for temporal variables (3, 31–34); wearable, smartphone, and wrist actigraphy studies support daily tracking (35–39).	Prospective associations support prognostic relevance, but do not establish mediation through autonomic–interoceptive changes or causal effects on transitions.
Emotion regulation and state outputs	Meta-analyses of emotion regulation in bipolar disorder, neuroanatomy of mania, emotional processing, and prefrontal–limbic network studies support the background of state outputs (4, 22–30).	State-related differences do not reveal transition mechanisms. Antecedent, concurrent, and consequential changes should be distinguished.

## 6 Clinical implications and study limitations

This model identifies hypothesis-generating research targets rather than current treatment recommendations. Future pilot studies could assess the feasibility of emotion labelling, bodily signal discrimination, and non-catastrophizing attribution training, and test whether these approaches modify prespecified measures of interoceptive processing and emotion regulation. Their effects on mood-state transitions and clinical outcomes would require subsequent controlled trials. The main limitations of the current evidence include the following: studies directly comparing objective interoception across different mood states remain scarce; HRV is significantly influenced by respiration, medication, physical fitness, and substance use; self-report scales cannot substitute for task-based indicators; predictive coding serves only as a theoretical organizing framework in this article and has not been validated; and transdiagnostic evidence cannot replace direct evidence in bipolar states (48, 49). In addition, substance use, including alcohol, is an important confounding variable (50). Furthermore, bipolar I disorder, bipolar II disorder, mixed features, and different medication statuses should not be assumed by default to represent the same physiological phenotype.

## 7 Conclusion

Current evidence supports associations among mood states, interoceptive processing, autonomic function, and sleep/circadian rhythms, with selected longitudinal studies providing prospective or directional evidence. Whether autonomic–interoceptive changes precede and causally contribute to mood-state transitions remains unresolved; the proposed framework therefore specifies hypotheses for longitudinal and experimental testing. The value of this framework lies in generating falsifiable longitudinal predictions and in explicitly noting the insufficient evidence for mania/hypomania, the non-equivalence of HRV and interoception, and the necessity of controlling for confounding factors. .
